# Spectrum Sensing for Cognitive Radio: Recent Advances and Future Challenge

**DOI:** 10.3390/s21072408

**Published:** 2021-03-31

**Authors:** Abbass Nasser, Hussein Al Haj Hassan, Jad Abou Chaaya, Ali Mansour, Koffi-Clément Yao

**Affiliations:** 1 LABSTICC UMR CNRS 6285, ENSTA-Bretagne, 29806 Brest, France; ali.mansour@ensta-bretagne.fr; 2 ICCS-Lab, Computer Science Department, American University of Culture and Education, 1507 Beirut, Lebanon; 3 CCE Department, American University of Science and Technology, 1100 Beirut, Lebanon; hhajhassan@aust.edu.lb; 4 Applied Physics Lab, Faculty of Science, Lebanese University, 1200 Jdeideh, Lebanon; jad.abouchaaya@ul.edu.lb; 5 LABSTICC UMR CNRS 6285, Université de Bretagne Occidentale, 29238 Brest, France; koffi-clement.yao@univ-brest.fr

**Keywords:** cognitive radio, channel sensing, Interference Sensing, spectrum sensing, full-duplex, half-duplex, internet of things, wireless sensor network, machine learning, 5G, B5G

## Abstract

Spectrum Sensing (SS) plays an essential role in Cognitive Radio (CR) networks to diagnose the availability of frequency resources. In this paper, we aim to provide an in-depth survey on the most recent advances in SS for CR. We start by explaining the Half-Duplex and Full-Duplex paradigms, while focusing on the operating modes in the Full-Duplex. A thorough discussion of Full-Duplex operation modes from collision and throughput points of view is presented. Then, we discuss the use of learning techniques in enhancing the SS performance considering both local and cooperative sensing scenarios. In addition, recent SS applications for CR-based Internet of Things and Wireless Sensors Networks are presented. Furthermore, we survey the latest achievements in Spectrum Sensing as a Service, where the Internet of Things or the Wireless Sensor Networks may play an essential role in providing the CR network with the SS data. We also discuss the utilisation of CR for the 5th Generation and Beyond and its possible role in frequency allocation. With the advancement of telecommunication technologies, additional features should be ensured by SS such as the ability to explore different available channels and free space for transmission. As such, we highlight important future research axes and challenging points in SS for CR based on the current and emerging techniques in wireless communications.

## 1. Introduction

Two decades ago, Mitola introduced a new concept in wireless telecommunication: the Cognitive Radio (CR) [1]. CR is mainly based on Soft Defined Radio (SDR) [2], where specific hardware can be replaced by more generic hardware that can be configured via software. In addition to being softly configurable, CR is aware and adaptable to the radio environment, which can be exploited in optimizing the use of available frequency bands while protecting the occupied ones from harmful interference.

Most of the current wireless communication systems are based on the concept of fixed (or static) frequency allocation. They are designed to operate on pre-selected frequency bands. This static allocation results in a low spectrum utilization especially at low traffic periods. Based on [3], the usage of some allocated frequency bands is lower than 15%. In addition, the development towards 5G and Beyond (B5G) technologies, the exponential growth in the number of connected objects via the Internet of Things (IoT), Wireless Sensor Network (WSN) devices and recent wireless applications push the wireless communication community to enhance the utilization of the limited frequency resources to satisfy the increasing demand on the wireless communication services [4,5].

**Figure 1:**
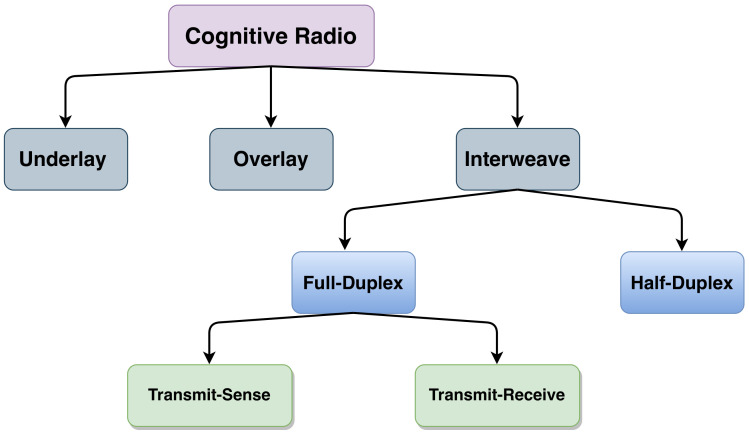
Cognitive Radio (CR) access paradigms.

CR has been introduced as a potential candidate to perform complete Dynamic Spectrum Allocation (DSA) by exploiting the free frequency bands that are aslo called “spectrum holes” or “white spaces” [6,7,8]. Being capable to identify these spectral opportunities, CR classifies the users into two categories: licensed, i.e., the Primary Users (PUs), and unlicensed, i.e., the Secondary Users (SUs). While PUs can access the spectrum whenever they want, SUs are restricted by the activities of PUs. In other words, SUs should respect the PUs’ Quality of Service and harmful interference coming from SUs to PUs transmission is prohibited. Therefore, three paradigms of CR can be distinguished according to the possibility of co-existence of SU and PU transmissions in the same channel, the permitted transmit power of SU and the cooperation between SU and PU. As shown in Figure 1, three main paradigms of CR can be distinguished:

1. Underlay Access: the SU may transmit simultaneously with the PU over the same channel. However, the transmitted power should not exceed a certain threshold in order to keep the interference on PU below a tolerable value [4,9].
2. Overlay Access: the SU may transmit simultaneously with the PU on the same channel up to its maximum power, but at the cost of playing a role of relay between two or more PUs [10,11]. In this case, the SU sends its data while relaying the PUs. This kind of access requires high level of cooperation between PUs and SUs, which may expose the PUs privacy.
3. Interweave Access: SU is allowed to transmit using its maximum power only when PU is absent. This paradigm is also known as the classical CR and it is the focus of this paper given its popularity.

The main drawback of the underlay paradigm is the low transmitted power, which adversely impacts the throughput. The use of the overlay paradigm is limited to scenarios where PU and SU have a high level of cooperation. Interweave paradigm allows SUs to transmit with their maximum power, but at the cost of monitoring the activity of PU.

In classical interweave systems, the SU activity period is divided into two time slots: sensing and transmission. This leads to the so-called Half-Duplex Cognitive Radio (HDCR). HDCR applies the Listen-before-Talk (LBT) protocol by adopting an alternating sensing-transmission fashion. During the sensing slot, the SU only senses the channel to detect the presence or absence of the PU, and it cannot transmit. SU should remain silent if it detects a transmission from the PU. Otherwise, the SU resumes sending its data. Note that the silence of the SU during the sensing slot affects its transmission rate. Moreover, periodic sensing may lead to collisions between the SU and PU as the PU may become active again during the SU transmission after being silent during the SU sensing slot.

In-Band Full-Duplex (IBFD) communication has recently been proposed in order to increase the spectrum efficiency [12]. Two peers can use the same frequency channel to transmit and receive simultaneously. IBFD is based on the Self-Interference Cancellation (SIC), where the Self-Interference (SI) is canceled in order to obtain the signal of interest. The application of SIC has been extended to CR providing it with the ability to sense and transmit at the same time leading to the so-called Full-Duplex Cognitive Radio (FDCR).

Based on the SIC capability and the flexibility of FDCR, several access schemes have been proposed and various challenges have been treated such as the SIC-based modes to be adopted, hybrid mode between HDCR and FDCR and the effects of the residual SI on the sensing process since SIC is imperfect.

As a powerful tool, machine learning techniques are exploited in the domain of CR to improve the SS performance [13,14,15]. SS may be formulated as a binary classification problem related to the presence of PU. Unlike classical SS, the learning techniques may overcome the need to know statistical parameters of the channel or the PU signal. Moreover, these techniques are proposed to predict the PU activity, which can enhance the spectral efficiency of the secondary network and protect the primary transmission from the secondary interference.

The usage of CR is extended to the domains of IoT and WSN [16,17]. This was motivated by the huge number of new IoT/WSN devices that require additional frequency resources. Although using CR for WSN and IoT seems promising, more investigation is needed at several levels such as the design of the exchange protocols and access management.

CR for fifth Generation (5G) is expected to play an important role to answer the need of the increasing number of data hungry devices [18]. Knowing that 5G will extend the spectrum band to the millimeter-wave range, CR can be used to improve the spectrum utilization while providing better protection to co-existing users. Moreover, CR can be used to address interference issues from space, frequency and time domain. This is important knowing that 5G is expected to exploit spatial reuse of the spectrum as one of the main features of 5G systems. Yet, introducing CR in 5G imposes several challenges that need addressing.

CR is proposed to be used in various wireless communication technologies, since it proves itself as one of the efficient techniques to ensure fair and flexible frequency allocation [18,19]. CR benefits from the emergence and development of learning techniques applied to wireless communication [20,21]. Accordingly, SS should be ceaselessly improved to keep up with the recent technological advancements. In this context, several challenges are raised such as the need for huge frequency resources, sensing of the spatial availability, intelligent sensing of the spectrum and energy-efficient protocol design.

In the literature, several papers survey the usage of SS for CR. In [22], the authors presented many aspects of spectrum sensing from a cognitive radio perspective. However, this paper is published more than 12 years ago and did not address the recent applications and paradigms. [23] surveys FDCR technique by focusing on the concurrent transmit–sense mode while other techniques, such as transmit–receive, were not covered. Ref. [24] details the challenges of applying CR in IoT networks by focusing on the issues related to SS. [25] surveys the techniques of SS with a focus on wideband and compressive sensing. In [26], the authors survey the recent techniques of SS by highlighting the mathematical models deriving the SS metrics (detection and false alarm probabilities). However, recent paradigms, such as Full-Duplex, and recent applications, such as the Internet of Things, are not addressed. The work in [27] is limited to technical issues related to the application of CR in IoT. Finally, the authors of [18] investigate the use of CR for 5G communication without further explanation of recent development in SS.

This paper aims at providing comprehensive surveying and analysis of the recent research advancements and emerging applications in the field of SS for CR. For numerical evaluation of SS techniques, the readers are encouraged to refer to [22,26]. We explain the fundamental concepts of SS and summarize the state of the art in the context of SS for CR. Moreover, we discuss the use of machine learning to enhance SS and the applications of CR in IoT/WSN from SS perspective. SS for 5G-based application is also discussed in this manuscript. Finally, we propose possible perspectives to develop this promising domain. The main contributions of the paper may be summarized as follows:

- A state of the art on the classical SS techniques is provided
- The operating modes of CR derived from involving the FD tool in CR are detailed and investigated
- The role of Machine and Deep Learning in enhancing the SS is surveyed, where we analyzed the contributions of these techniques from local sensing and cooperative sensing levels
- Using SS in IoT/WSN and the latest achievements in both Spectrum Sensing as a Service and Dynamic Spectrum Sharing for IoT/WSN networks are surveyed.
- The possible application of CR, especially SS, in the 5G and the upcoming technologies is discussed
- New trends and challenges related to the future wireless communication technologies are also discussed and investigated.

The rest of this paper is organized as follows. Table 1 presents the list of abbreviations used in this survey. In Section 2, HDCR is presented by explaining classical SS using the traditional detection methods. The silence period of HDCR is also discussed in detail at both levels of SU throughput and collision to PU. FDCR is described and the related operating modes, as well as the sensing process, are discussed in Section 3. In this section, we present the main techniques behind the FDCR. Then, we present the popular derived modes using SIC, Transmit-Sense and Transmit-Receive with thorough analysis and investigation. The application of learning techniques for SS is explained in Section 4. Section 5 surveys the latest achievements of the CR applications in IoT/WSN networks. The role of IoT/WSN in applying Spectrum Sensing as a Service, and the CR-based operation of IoT/WSN are also considered and analyzed. A discussion on the use of spectrum sensing for 5G-based CR applications is presented in Section 6. Section 7 presents several future challenges related to applying and developing SS in several domains, such as: IoT/WSN, the CR operating modes, the future technologies, the access strategies of SU, the use of emerging techniques, and recent smart SS. Finally, Section 8 concludes our manuscript.

## 2. Half-Duplex Cognitive Radio: Listen Before Talk

Listen-Before-Talk (LBT) protocol consists of applying the SS periodically [22,28]. The activity period of each SU in LBT is divided into two slots: sensing and transmission. During the sensing slot, SU silently performs the SS to avoid affecting its decision reliability. LBT refers to the so-called Half-Duplex Cognitive Radio (HDCR) due to this silent period of transmission. If the PU is absent, SU switches to the transmission slot and transmits its data without being aware of the PU activity. The received signal under HDCR can be presented as follows [22,29,30]:

(1) y(n)=ηx(n)+w(n),

where y(n) is the received signal, η is the channel indicator, i.e., η=0 if PU is absent and η=1 otherwise. x(n) is the PU signal and w(n) is the additive noise at the receiver of SU. For active PU (η=1), SU receives a noisy version of the PU signal. Therefore, it should be aware of the PU channel status by overcoming the noise effects. By contrast, when PU is absent, SU receives only the noise w(n). Here, SU should be able to detect the presence or the absence of PU to better exploit the channel availability. In Equation (1), the received signal y(n) does not depend on the SU signal, since we consider that SU remains silent during the SS period.

The silence period of SU in HDCR is inevitable due to two main challenges. First, some detectors like Energy Detector (ED) cannot distinguish between PU and SU signal. Thus SU must be silent during the sensing period so that the sensing process can reliably diagnose the channel status. Second, most of the detectors suffer bad performance at low Signal-to-Noise-and-Interference Ratio (SNIR). Due to the short distance between the transmit and the receive antennas of SU, any transmission of SU during the sensing period leads to huge SI compared to the PU signal. This SI would lead to an unreliable decision of the sensing process.

### Detection Criteria

To detect the PU signal, SU evaluates a Test Statistic based on the received signal y(n). The aim of evaluating TS is to compare it to a threshold to decide on the PU status by distinguishing between the noise-only case and the PU-plus-noise case. Many criteria have been exploited to distinguish between these two cases. In the following, we discuss the most adopted criteria that are used by the SU to detect the presence of PU.

1. Incremental Energy
  When PU starts to transmit, the energy of the received signal will be incremented compared to the noise-only case. By estimating previously the power of the stationary noise, and by comparing the energy of the received signal to a pre-defined threshold depending on the noise power, SU decides whether the channel is occupied by a PU signal or not. Many detectors are based on this criterion, the most known is the traditional ED [30,31]. Other detectors such as the Cumulative Power Spectral Density (CPSD) detector [29], cyclo-energy detector [32] and generalized ED [33,34,35] are based on differentiating between the energy of the received signal with and without the presence of PU’s signal. It is worth mentioning that the generalized ED may use a power exponent p≠2 in the definition of it as an extension of the ED Test Statistic, which is based on the energy of the received signal (i.e., p=2). However, the energy-based detectors face the problem of Noise Uncertainty (NU), which occurs when the noise power becomes time-dependent. This phenomena adversely impacts the SS performance of these detectors [36].
2. PU signal pattern
  The features of the communication signals can be exploited by the SU to distinguish them from the noise. Processes, such as the modulation, oversampling, sine-wave carrier, adding a cyclic prefix (e.g., for the OFDM signal), etc. do not exist in the noise. Several detectors were proposed in the literature by exploiting these characteristics such as the Cyclo-stationary Detector (CSD) [37,38,39], which distinguishes the PU signal from the noise based on the cyclic features caused by the modulation, the sinewave carrier etc. Other detectors such as Auto-Correlation Detector (ACD) [40] and Eigenvalue-based Detector (EVD) [41] exploit the correlation presented in the PU signal due to the oversampling and cyclic prefix. The main advantage of such detectors is their independence of the noise variance, which also overcome the NU problem. Nevertheless, these detectors are more computationally complicated than the classical ED. Furthermore, cyclic frequencies of the PU signal should be known to apply CSD. This requires cooperation between SU and PU.
  Moreover, some detectors, such as Goodness of Fit (GoF) test [42,43] and Kurtosis detectors [44], detect the PU signal using the statistics of the communication signals, which are different from the statistics of noise. Thus, the noise’s distribution should be a priori known to the SU.
3. PU signal’s waveform
  Sending a pilot signal is widely used by telecommunication standards to establish communication with a receiver by ensuring time synchronization, channel estimation, etc. A known PU pilot signal can be used by the SU to detect PU activity. Waveform or Matched filter detector correlates the received signal with the known PU pilot signal in order to analyze the channel opportunity [45,46]. Even though this detector is an optimal one, it requires knowledge of the PU signal with perfect time and frequency synchronization. Therefore, the application of this detector in CR becomes challenging, where the SU may deal with a great variety of signals.

Table 2 compares among several SS detectors with respect to the impact of the noise uncertainty, the need of cooperation between SU and PU and the computational complexity.

As shown in Table 2, the choice of a detector depends on several factors such as the PU-SU cooperation, the computational cost of the detection method and the precise estimation of the noise variance. Other criteria can be considered such as the required observation time (i.e., the number of received samples). A long observation improves the SS performance, but consumes more energy and reduces the spectral efficiency of SU, since the latter should be silent during the sensing operation. In contrast, a low observation time may increase the collision rate between SU and PU, since a low number of received samples may not be sufficient for the detector to reveal the true channel status. Thus, choosing the observation time is a trade-off between the spectral efficiency and the collision rate. Furthermore, choosing a period between two sensing operations (i.e., transmission period) is also challenging in HDCR. On the one hand, short periods ensure low collision time with PU, but also reduce spectral efficiency and increase energy consumption due to the sensing operations. On the other hand, long periods allow SU to consume less power by SS and increase the spectral efficiency but at the cost of a long colliding time with PU. In brief, two major problems of LBT could be listed:

1. The SS is not performed during the transmission slot, and thus the SU becomes unaware of the PU activity during this slot. This may lead to harmful interference with PU if the PU starts transmitting in this slot.
2. The secondary throughput is affected by the silence duration (sensing time), since the SU should stay silent during the sensing slot.

These two problems can be solved by FDCR as explained in the next section.

## 3. Full-Duplex Cognitive Radio: Listen and Talk

**Figure 2:**
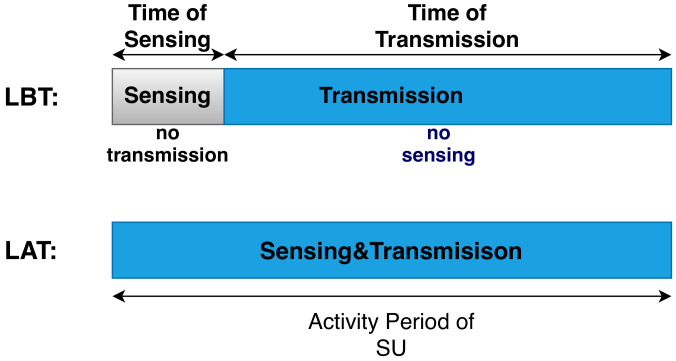
The two main functioning modes of SU activity. Listen-before-Talk (LBT): SU remains silent during the sensing period and no sensing is performed during the transmission. Listen and Talk (LAT): sensing and transmission are made concurrently.

Applying SIC in CR aims at eliminating the effect of the transmitted secondary signal (i.e., Self-Interference) on the reliability of the SS decision. SU seeks to receive purely the PU signal (with noise) when the latter is active, or only the noise if PU is absent. Thus, the transmitted signal of the SU should be canceled at the SU receiving antenna, if the SU starts to simultaneously transmit and sense. As shown in Figure 2, by applying LAT protocol, FDCR can continuously monitor PU without the need to interrupt the transmission to make the SS, as in the case of HDCR where LBT is applied.

### 3.1. Self-Interference Cancellation

The key technology behind the IBFD communication, SIC, can be divided into two steps: passive cancellation and active cancellation. In passive cancellation [53,54,55,56], the SI is canceled in the analog domain considering several parameters such as the distance between the transmit and the receive antennas, the wavelength of the signal, the absorption of the material, etc.

In active cancellations [57,58,59], the receiver suppresses the SI in the digital domain given that the receive and transmit circuits are co-located and that the SI signal is known at the receiver. Due to the short distance between the transmit (TX) and the receive (RX) antennas, the SI received power at RX is huge compared to the signal of interest (PU signal). Thus, the channel estimation between TX and RX should be very precise in order to re-generate the SI with high precision and to cancel it afterwards. Moreover, the hardware imperfections adversely impact the SIC performance and should be mitigated, since their power becomes very high compared to the signal of interest. Note that the hardware imperfections are due to several factors including oscillator phase noise, the non-linearity of the amplifiers, the ADC noise, etc. Subsequently, due to the error in channel estimation and the hardware imperfections, Residual Self-Interference (RSI) remains at the receiver.

RSI is modeled as both linear and non-linear combinations of the SI signal due to the amplification at both the output of the transmit circuit and the input of the receive circuit [60]. Mitigating these imperfections has gained a lot of attention during the last decade especially the multiplicative noise of the oscillator and the non-linearity of the power amplifiers [59,61,62,63,64]. In the following, we present the modes of operation of FDCR.

### 3.2. Transmit-Sense

Transmit-Sense (TS) mode is a direct result of applying the LAT protocol. SIC is used to cancel the SI when the SS is performed. Thus, the sensing performance is affected by the SIC efficiency. In the literature, several models have been adopted to represent the received signal considering the impact of the RSI on the sensing performance [31,58,65,66,67]. Among the most used, we consider the following [66,67,68]:

(2) y(n)=Es(n)+ηx(n)+w(n)

where s(n) is the SU signal received at RX, including the channel effect and the hardware imperfections; E is the SIC efficiency, where 0≤E≤1; When E=0, then the RSI suppression is perfect while E=1 corresponds to the case of no RSI suppression.

After canceling the SI, a Test Statistic is applied on y(n). Usually, the Test Statistics used in HDCR can also be used for FDCR. As shown by Equation (2), the received signal in FDCR mode becomes the same as that of HDCR for ideal SIC (Equation (1)). Therefore, the SS performance under HDCR becomes an asymptotic case for FDCR [68]. The last statement is true for the case of a single SS operation. However, FDCR can continuously perform SS while sensing in HDCR is not applied during the transmission period of SU. Knowing that the PU can become active at any time, the collision rate with PU is highly reduced in FDCR compared to HDCR [69].

**Figure 3:**
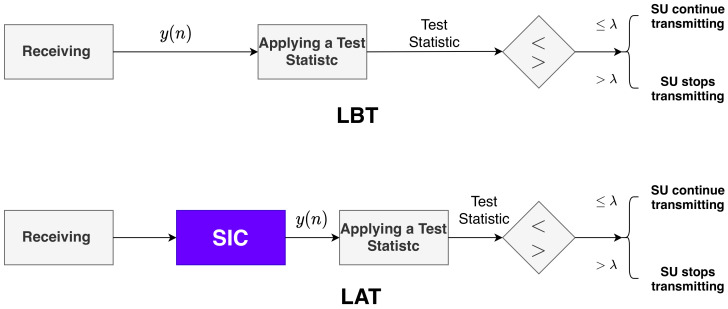
Spectrum Sensing processes under LBT and LAT. For LBT, no Self-Interference Cancellation (SIC) module is required since there is no simultaneous Transmit-Sense (TS), and the Test Statistic may be directly applied to the received signal. By contrast, SIC is applied before evaluating the Test Statistic in LBT to reduce the effect of SI on the Spectrum Sensing (SS) performance.

Figure 3 shows the steps of FDCR and HDCR before making a decision on the channel state. After receiving the signal, a Test Statistic is evaluated in HDCR, while SIC precedes the Test Statistic evaluation in FDCR in order to reduce the SI effect on the reliability of the Test Statistic.

Another strategy for the LAT protocol is adopted in [70] without using SIC. The sensing operation is performed by the receiving SU instead of the transmitting one. After decoding the signal received from its peer (another SU), the secondary receiver subtracts the signal of its peer from the overall received signal. After that, spectrum sensing is carried out based on the remaining signal in order to decide about the presence/absence of the PU.

From another perspective, SIC necessitates additional hardware requirement. Auxiliary chain is mandatory in SIC-based receivers in order to maintain the synchronization with the transmitter and to extract some necessary features to reduce the SI [58,62]. The additional hardware equipment increases the cost and the required power of the FDCR system compared to the HDCR [66,71]. Moreover, making the SS continuously may negatively impact the energy efficiency of the system due to the additional power consumed by the SIC circuit and the SS itself [72]. As a compromise, the period between two consecutive SS operations is configured based on the accepted energy efficiency of the system and the tolerable collision time with PU. Another parameter impacting the period between two consecutive SS operations is the probability that the PU returns active [73,74]. When the PU is more likely to be absent for a long time, continuous monitoring is not efficient.

### 3.3. Transmit-Receive

Transmit-Receive (TR) allows two SUs to establish bidirectional communication over the same channel. In this mode, SIC is used to cancel the SI in order to obtain the signal of the peer SU, unlike TS where SIC is used to receive the noisy primary signal when PU is active. The PU signal sensing is performed by Interference Sensing (IS) or SS as we explained in the following.

#### 3.3.1. IS-Based Transmit-Receive

Even though the SS is not performed by SUs in IS-based TR mode, PU detection remains applicable by employing IS instead of SS. IS is based on the capability of the secondary receiver to decode the message of its peer. When PU becomes active, it causes interference to the secondary transmission resulting in the inability of the second receiver to decode the message of its peer. Thus, if the decoding process is successful, the PU is assumed absent, while PU is detected as active otherwise. The main advantage of TR is that it doubles the spectrum efficiency since the same band is used for both transmitting and receiving data. However, it exposes the PU to a high risk of harmful interference as IS is not efficient at low PU’s SNR [75].

**Figure 4:**
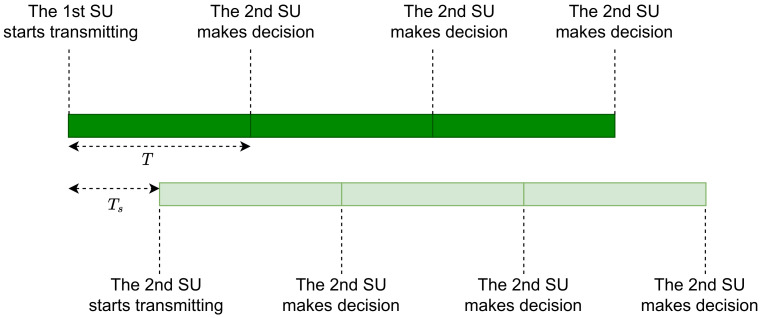
The communication mechanism in asynchronous Transmit-Receive (TR) mode. A delay of $T_{s}$ is made in order to reduce the collision time between the secondary and the primary transmissions.

Before the secondary system starts with the TR mode, it makes a traditional SS period in order to detect the vacancy of the primary band. This SS operation lasts while the PU band is occupied [65,76,77,78]. Once the PU band is identified as vacant, SUs start communicating using IBFD. In order to minimize the interference to the PU, an asynchronous transmission was proposed [65,76,77]. In asynchronous transmission, one of the two communicating SUs makes a delay of $T_{s}$ with respect to the transmitted frame of its peer. As presented in Figure 4, the decisions made by SUs at the end of each frame are delayed by $T_{s}$ with respect to each other. For an optimal value of $T_{s}=\frac{T}{2}$ [65], the CR network makes a decision on the PU status each $\frac{T}{2}$ sec instead of *T* sec if synchronous transmission is adopted between SUs. This asynchronous transmission helps the SUs to enhance monitoring the PU activity as the latter may access the channel at any time.

The application of IS-based TR provides SU with high spectral efficiency compared to TS. The main drawback of this technique is its weak awareness of the PU presence when the latter returns active with a low SNR. Therefore, it is recommended to use the IS-based TR in the scenarios where PU SNR is high at the SUs, so that it prevents SU receivers from decoding the messages when active.

#### 3.3.2. SS-based Transmit-Receive

Even though TS and IS-based TR are the most popular schemes in FDCR, another TR mode based on SS has been introduced. Performing SS in TR faces the problem of receiving the secondary signal of the peer SU in addition to the SI of the SU. The SIC is capable of highly reducing the SI signal. Yet, the signal of the peer SU still exists. The received signal in SS-based TR is given as follows [75,79]:

(3) y(n)=Es(n)+ηx(n)+r(n)+w(n)

The variables in Equation (3) are similar to those in Equation (2), except for the r(n) that stands for the peer SU signal including the channel effect.

In [79], a mechanism is proposed to apply SS-based TR that exchanges the sensing parameters between the two communicating SUs. When SU1 sends the signal to SU2, it sends concurrently the energy amount of the transmitted samples to its peer via a control channel. On the other side, SU2 computes the energy of the received samples and subtracts the known amount of the energy of its peer. However, this mechanism requires an accurate estimation of the channel between the communicating SUs. The proposed mechanism may also fail to detect the PU when the SU SNR is high.

The work presented in [75] aims to overcome the problem of PU’s low SNR that faces the TR mode by adopting SS instead of the IS. One of the two communicating SUs should remain silent only during the SS period in order not to disturb its peer. Hence, the sensing operation is performed in an alternative manner between the two communicating SUs. When the first SU performs the SS, it continues transmitting and canceling its SI. Meanwhile, the second SU remains silent. This mechanism is regularly alternating between the two SUs.

In [80], SS-based TR is proposed for OFDM-based FDCR. Two communicating SUs should avoid the use of some dedicated sub-carriers (null sub-carriers) in order to detect the PU status. When the PU becomes active, it might cover the entire band including the dedicated sub-carriers. As these sub-carriers do not exhibit any secondary transmission, SU may monitor PU by evaluating the energy of the received signal on these sub-carriers. Note that no SIC is required to be done on the sub-carriers where the SS is applied since no secondary transmission is made on them. The cost to pay in this SS-based TR mechanism is the loss in the frequency resources, i.e., the null sub-carriers.

Table 3 presents a comparison among the CR operating modes with respect to the essential features related to the SU performance and the impact of SU’s transmission on PU. The IS-based TR presents a poor performance at low PU SNR in contrast to other modes. HDCR may lead to a long collision time with PU due to its blindness during the transmission, while FDCR modes can perform the sensing continuously. The need for SIC to perform the SS exists in TS mode and some approaches in SS-based TR mode. Note that SIC can be used for the sake of establishing bidirectional communication between the peer SUs in TR modes.

## 4. Learning Techniques for Spectrum Sensing

Machine Learning (ML) and Deep Learning (DL) are among the most powerful tools in solving complex classification problems. More specifically, they have been employed in wireless communication to efficiently manage the spectrum and the power resources, and to ensure high quality of services for the mobile users [84,85,86,87,88]. In the CR domain, one of the objectives of using ML and DL is to enhance the SS performance. Learning techniques usually use two phases: learning and prediction. For SS applications, the data provided in the learning phase is related to the PU features and the SU sensing parameters (such as the Test Statistics, SNR, geo-location, etc.), whereas the prediction phase could be related to the sensing outcome, the power efficiency, the functioning model to be adopted and other issues [89].

In classical SS, SU has to determine the threshold for the Test Statistic before making a decision on the PU presence. This threshold may be calculated based on target false alarm and detection rates. Thus, several statistical parameters related to the noise, the channel, and the PU signal should be a priori known. ML and DL can overcome the need for the a priori statistics knowledge [13,90,91,92,93,94]. In literature, the majority of work focuses on tuning ML or DL systems with numerical statistics of two hypotheses: $H_{0}$, where PU is assumed to be absent, and $H_{1}$, where PU is assumed to be active.

Hereinafter, we discuss the use of learning techniques in local and cooperative spectrum sensing.

### 4.1. Local Spectrum Sensing

Local sensing corresponds to the case where a single node senses the spectrum and makes its own decision. In this context, the work of [95] seeks to discriminate between $H_{0}$ and $H_{1}$ hypotheses by being trained with the extracted cyclic features of PU’s signal in low SNR conditions. This ensemble classifier is based on decision trees and using AdaBoost algorithm [96]. Wideband SS is tackled in [97], where three techniques are presented: neural networks, expectation maximization and k-means. The techniques are used to detect the presence of one or multiple PUs in a wideband spectrum.

In order to enhance the accuracy of the ML system in making a decision on the PU status, Hybrid SS (HSS) has been proposed [92,98]. HSS detects the presence of PU using simultaneously several detectors. HSS can compensate the weak points of a given detector with the advantages of another one. For instance, ED suffers from the noise uncertainty at low SNR, which is overcome by ACD. In return, ACD is adversely impacted by the low oversampling rate of the PU signal, while ED is not [29].

In [92,98], artificial neural networks have been applied in order to perform HSS. Training is done using the Test Statistics of two detectors related to $H_{0}$ and $H_{1}$, where ED and CSD are used in [98], while ED and likelihood ratio statistics are used in [92]. The work of [99] extends the HSS-based DL for 6 detectors showing the effectiveness of such techniques in detecting PU at very low SNR. HSS is exploited in [100] in order to introduce one-class-based learning. Data of several detectors are collected under $H_{0}$ to learn the detection system of the $H_{0}$ class. The training phase is done using only data related to the noise without the need for PU-related data. In the prediction phase, when the predictor detects an outlier of $H_{0}$ class, then PU is assumed to be active.

However, these techniques, especially those related to DL, have a computational cost. Some work deals with the trade-off between accuracy and computational complexity when using DL for SS in order to ensure high energy efficiency such as in [101,102].

Furthermore, ML and DL are used for spectrum perceptions, i.e., prediction of the occupancy of the PU channels [89,103,104]. For example, a geo-frequency-temporal map on the PU activities can be constructed using learning techniques. This map guides the spectrum access and enhances the SS performance [105]. Using the prediction of the spectrum vacancy, SU can wisely select the communication channels to reduce handover rate and avoid interference [73,106,107]. By contrast, when a handover happens, SU may target a channel where PU is expected to be absent to make SS before accessing [108]. This makes the handover safer and faster. Moreover, when PU is likely to leave the channel for a long time, SU may increase the period between two consecutive SS operations leading to economizing its energy and enhancing the spectral efficiency. The channel prediction may differ from a geo-location to another [109]. Accordingly, a mobile SU would switch from a channel to another based on its prediction of the PU behavior. In addition, the learning techniques proved their effectiveness in managing the priority of the SUs in accessing available channels. The SUs may be competitors or cooperatives in sharing the limited frequency resources. Thus, an efficient accessing policy is required in order to fairly distribute the available frequency resources over the SUs [110,111,112].

### 4.2. Cooperative Spectrum Sensing

In Cooperative SS, several SUs cooperate in order to make a final decision on the PU state. Two schemes of Cooperative SS can be distinguished [113,114,115]:

(1) Hard Decision Scheme, where each SU makes an individual decision on the PU state, then decisions of all SUs are combined at a Fusion Center (FC) to outcome a final decision.
(2) Soft Decision Scheme, where the FC gathers the Test Statistics calculated at the SUs, and combines them in order to compare a final Test Statistic to a threshold and make a decision on the primary channel.

ML and DL are introduced in cooperative SS in order to tackle several problems such as the correlated results between the cooperating SUs, the malicious results provided by some SUs, giving the SUs close to PU more credibility over far SUs, and other issues. In [91], ML techniques such as the K-Means and Support-Vector Machine (SVM) are used to distinguish between the $H_{0}$ and $H_{1}$ hypotheses in a cooperative SS. Two low-dimension probability vectors related to both $H_{0}$ and $H_{1}$ of ED are used to train the system. SVM is used to set the threshold curve between $H_{0}$ and $H_{1}$ clusters. K-nearest-based ML is adopted in [116] for cooperative SS, where the proposed mechanism is divided into two phases: training and classification. The global decision of the PU presence/absence taken at the end of the classification phase takes into consideration the reliability of each CR user when reporting to the fusion center during the training phase.

A convolutional Neural Network-based cooperative SS is proposed in [15], where the outputs of the SUs are combined in Hard and Soft Combining Schemes. Spatial and spectral correlations of the channels are taken into account in order to make the system more robust. The SUs, which are close to each other, may report correlated decisions to the Fusion Center. This negatively impacts the sensing performance when these SUs exhibit severe fading [117].

The authors in [20,101] propose using the learning techniques for cooperative SS in a non-orthogonal multiple access context in order to overcome the physical layer complexity of such access scheme. Reinforcement learning is adopted in [118] to perform the cooperative SS. The reinforcement learning scans the PU channels to form a dynamic scanning preference list. This list helps to reduce the scanning overhead and access delay.

Using learning techniques in spectrum monitoring for CR is still open to investigation. The switching among the channels to sense, the switching between functioning modes (TS, TR, HDCR, etc.), the sensing rate, and other features are to be tackled by learning techniques.

Table 4 classifies the recent works that apply learning techniques in CR into four classes: local SS, cooperative SS, spectrum prediction, and resource management. Papers focusing on more than one topic are also considered, such as local SS and prediction, and local SS and resource management. The mark ✓(resp. ✗) means that the subject is covered (resp. is not covered) by the mentioned papers.

## 5. Wireless Sensor Network and Cognitive Radio

The use of WSN/IoT is substantially expanding, and it is expected to cover almost all of the life sectors: monitoring purpose, traffic, e-health applications, smart homes, agriculture, etc. CR and WSN/IoT can significantly benefit from each other. On the one hand, the wide deployment of WSN/IoT can be exploited by the CR in monitoring the PU channel. For example, Spectrum Sensing as a Service (SSaaS) emerges as a new business model [142]. On the other hand, the huge number of WSN/IoT devices give rise to high demand on spectral resources. Here, CR technology can be considered as a solution thanks to its dynamicity in enabling spectrum sharing [16,17].

### 5.1. Spectrum Sensing as a Service

When considering SSaaS, the SS process is done by WSN node(s) related to the SSaaS provider and not by the SU in the secondary network. The SSaaS provider then informs the secondary network with the PU channel status [142]. Accordingly, the secondary network takes the decision on transmitting on that channel or not. SU may operate in a wide range of frequency channels, therefore, WSN should have the necessary electronic circuitry to reconfigure according to the sensing tasks requested by the secondary network.

Moreover, on-demand responsiveness of the WSN network is of high importance, since the SS information on the PU channel should be relevant, and SU should be up-to-date continuously [143]. This challenge faces the limitation associated to the WSN and the IoT networks protocols such as LoRaWAN, since such a network does not support the on-demand data communication [144]. The work of [16,145] suggested some modifications on LoRaWAN protocol to support the on-demand SS.

The selection of the WSN nodes that perform the SS is also a serious challenge due to the energy constraint imposed by the massive range of battery-powered nodes. Several strategies may be adopted to select the nodes responsible for performing the SS: random-based selection, SNR-based selection, battery’s energy level-based selection, hybrid criteria-based selection [145,146,147,148]. Each of the strategies has its impact on the SS performance and on the lifetime of the network. For instance, SNR-based strategy ensures high SS performance but it may adversely impact the lifetime of the nodes. By contrast, the battery’s energy level-based selection does not take into consideration the SNR of the primary signal, which may lead to poor SS performance.

From another perspective, it is important to study the contract between the secondary network and SSaaS provider to ensure the satisfaction of both entities [149,150,151]. In this context, blockchain technology is used to set the required SS parameters via smart contract, such as in [149,150], where the nodes of the WSN are rewarded only if they accurately perform sensing.

SSaaS remains an open research topic to explore. Several parameters are not studied yet, such as the impact of the delay of sending the sensing results from the SSaaS provider to the secondary network on the collision rate between SU and PU. In addition, the cost of SS in terms of the payment and the collision rate has to be further investigated. Large number of SS operations leads to high protection of PU against secondary interference, but it may be more expensive for the secondary network. In addition, it is important to study mobile SU in the context of SSaaS, where the mobile SU might leave a WSN to another. This will require handover execution in order to keep the SU aware of the PU activity. In addition, the SU needs to switch to a new vacant channel if PU returns active. Here, an efficient and fast cooperation strategy between the SSaaS provider and the SU network should be designed in order to find an available channel and to switch smoothly from one channel to another.

### 5.2. Dynamic Spectrum Sharing for WSN communication

The increasing popularity of using WSN/IoT devices requires better utilization of the limited frequency resources. Dynamic Spectrum Sharing using CR has been proposed to overcome the limitation of the available frequency resources in the context of WSN [17,24].

In Dynamic Spectrum Sharing for WSN communication, CR considers the WSN nodes as SUs. By monitoring the PU channel, the WSN nodes bear extra energetic burdens, since more energy consumption is needed to accomplish the SS task. In fact, energy consumption is of high importance for the IoT/WSN networks, since the nodes are operating under protocols to extend their lifetime to several years. For instance, in Low Power Wide Area Network (LPWAN) IoT networks, the lifetime of a sensor may exceed 5 years [152] due to the transmission specifications, especially the small duty cycle of less than 1% as in LoRa networks [153].

Adapting the CR mechanism to the WSN/IoT networks is challenging due to several issues such as providing the nodes with the available frequency bands, deploying the SS capability, selecting the nodes responsible of spectrum sensing, etc. Several studies tackle the implementation of CR in an IoT network and the standardization of the CR-based IoT [17,24,154,155,155]. They explain the additional features required to perform the CR mechanism such as implementing hardware and protocols. This includes switching from one frequency band to another depending on the sensing process. Indeed, multiband access becomes necessary due to a large number of WSN/IoT devices, and having several available frequency channels makes the channel switching simpler and more efficient [156,157].

The energy-throughput optimization of the CR-IoT is investigated in [158] where cooperative SS is adopted. Cooperative sensing necessitates the participation of several SUs/nodes in the SS. Despite the sensing improvement, cooperative sensing may impose additional energy consumption challenges for the participating SUs related to the continuous SS and reporting operations. The adoption of local sensing or cooperative sensing is discussed in [159] under different sensing conditions and applied to CR-based NB-IoT technology with slotted-ALOHA protocol. The study of [159] concludes that it is more convenient to use local SS when SNR is relatively high and limits the use of cooperative SS to the situations of low SNR.

Given that the CR functionality is implemented, new challenges emerge such as increasing the throughput and the spectrum efficiency and reducing the energy consumption of the wireless nodes. Dynamic licensed-unlicensed access is proposed in [16], where the CR-based nodes may access both licensed and unlicensed bands in a dynamic mechanism. Such a mechanism may lower the demand on the unlicensed bands (where the WSN/IoT nodes operate usually) by sharing available licensed bands.

In [158], an energy-throughput trade-off of wireless sensor network is proposed in the context of cooperative SS and dedicated low-power devices. The latter study focuses on minimizing the consumed energy while satisfying the requirements of secondary throughput and primary signal detection.

To compensate the additional energy consumed by the CR functionalities, such as SS and spectrum sharing, Energy Harvesting has been proposed in order to enhance the energy efficiency of the CR-based IoT system [160].

CR seems a promising mechanism to address the high demand of the WSN/IoT networks on the frequency resources. Yet, CR-IoT should prove its energy efficiency as well as the spectral efficiency especially for industrial applications [161]. The deployment pattern and the transmission protocol play an essential role to minimize the energy consumption and reduce the interference between nodes [162] leading to enhance the energy efficiency of the CR-IoT network. However, several challenges are to be investigated such as suitable protocol to exchange the data in two ways: central entity-sensing SUs and central entity-transmitting SUs. First, the central entity should inform the sensing SUs of the channel to be sensed. Then these SUs should respond back by their decisions. This mechanism imposes time and frequency synchronization, e.g., control channel. Second, after identifying the available channel, the SUs/nodes, which need to transmit, should be informed by adequate available channels to configure their circuit accordingly. This configuration necessitates the proper electronic circuitry that is able to meet the dynamic reconfiguration. In addition, efficient resource management by the central entity is required to fulfill the node demands in terms of data transmission and interference reduction.

## 6. Cognitive Radio Application for 5G and Beyond 5G

The 5th generation (5G) of mobile technologies is expected to provide users with Gbps communication, very low latency and high reliability. 5G networks are designed to operate on two types of channels: the licensed channels and the unlicensed channels [19].

### 6.1. 3GPP Technologies

Reaching 5G requirements is challenging especially those related to the massive connectivity and the Ultra-Reliable and Low Latency Communication (URLLC) [163]. CR is a potential candidate to help to enhance the utilization of the spectrum while protecting users in dense and heterogeneous networks [164].

In Release 13 of the 3GPP, LTE are provided with the ability to operate on the unlicensed band such as 2.4 GHz and 5.8 GHz in order to open new spectral resources [165]. Two types of unlicensed LTE can be distinguished: LTE-Unlicensed (LTE-U), developed by LTE-U Forum and used in USA, Korea and India, and LTE-Licensed Assisted Access (LTE-LAA), developed by 3GPP and used in Japan and Europe [166]. The use of unlicensed LTE is envisaged to extend to essential 5G applications such as the enhanced mobile broadband (eMBB), massive machine-type communication (mMTC), and URLLC [167]. LTE-U and LTE-LAA may cause severe interference to the technologies operating on the unlicensed bands, especially WiFi. Thus, an efficient mechanism is essential to manage the resource allocation and to protect the technologies from interfering with each other [168,169].

To protect WiFi against LTE interference, both LTE-U and LTE-LAA adopt Dynamic Channel Selection (DCS) by targeting the least interfering channels of the unlicensed bands to transmit over them [170]. However, DCS is not always applicable since no clean channel may be available. LTE-U uses Carrier Sensing Adaptive Transmission (CSAT), which is based on observing the channel for a certain time (usually between 0.2 to 10 sec) in order to define a time cycle. Then, LTE-U system transmits in a fraction of the cycle and turns the transmission off in the remaining duration [171]. Besides, CSAT, LTE-LAA uses LBT to access the channel [172,173]. Even though LBT is a SS technique and is applied in CR, more developed CR-based mechanisms have been proposed to be adopted by the unlicensed LTE in order to protect WiFi from the interference [168,174,175]. In such models, WiFi nodes are considered as PUs while the LTE-U users are considered as secondaries. In [175] a CR-based framework is proposed to construct the spectrum availability map. Idle LTE nodes perform SS on the unlicensed channels and send the channel status to the LTE base station periodically. Accordingly, the WiFi access-points locations and transmitting powers can be obtained and exploited later on to serve LTE-U. Joint spectrum sharing and aggregation is proposed to utilize the TV white spaces, licensed spectrum and LTE-U bands [176]. TV and WiFi systems are considered PUs in the TV white spaces and the LTE-U bands respectively. A co-existence strategy is developed to fairly utilise and access the TV and the LTE-U bands based on the sensing capability of the LTE network. In [177,178], sensing is used to reduce interference in ultra-dense small-cell deployment scenario, which introduces CR as a helpful technique for the planning of 5G and B5G networks.

### 6.2. Compressive Sensing

With the existence of various applications that require high spectral resources in 5G such as smart cities, smart agriculture, monitoring purpose, etc, the need for frequency resources becomes extremely high. This need will continue growing with the coming of the B5G and the Internet of Everything (IoE) [179,180]. In this regard, SS that focuses on one narrow channel, such as ED and CSD, may not be sufficient. Instead, Wide Band Sensing (WBS) [181,182,183], which is able to explore a wide frequency band, will be a good candidate to meet the frequency needs of the CR network. From the CR point of view, WBS ensures a high data rate for the SUs since the data rate is directly related to the bandwidth. In addition, WBS provides CR with the ability to satisfy multiple SUs requirements at the same time. A large available band for a CR makes the handover operation simpler and faster.

However, WBS imposes several challenges at the hardware complexity level [184]. In classical WBS that respects the Nyquist rate, the wideband is divided into a set of narrowband channels using filtering blocks. Then, a SS technique is concurrently applied on each channel to diagnose its availability. Despite the low delay in making the decision on the channels’ availability, this approach is very costly from a hardware point of view since it needs high filtering capability [185]. As an alternative, wavelet techniques have been proposed to perform the WBS. They are used to detect the irregularities of the wideband, in which the available channels are located. However, this approach suffers high computational complexity [186,187].

To overcome the Nyquist rate constraint, Compressive Sensing (CS) has been proposed to perform the WBS [181,188,189]. CS consists of three phases: sensing measurements, signal reconstructing, and finally performing SS on the reconstructed signal.

Recently, CS gained a lot of attention as in the case of joint communication and radar sensing in 5G mobile networks [190,191]. As for the SS, the use of CS focuses on the IoT applications, where the number of devices is huge [159]. Although CR solves the problem of the high sampling rate, it may suffer from the NU problem at the performance level [192], and power and complexity limitations at the implementation level. Low-cost systems and battery-powered devices, such as the IoT devices, may not afford the high complexity and hardware requirements of CS. For this reason, many studies focus on analyzing and lowering the cost of applying the CS in IoT networks [27,193,194,195].

### 6.3. Beamforming-Based Communication

**Figure 5:**
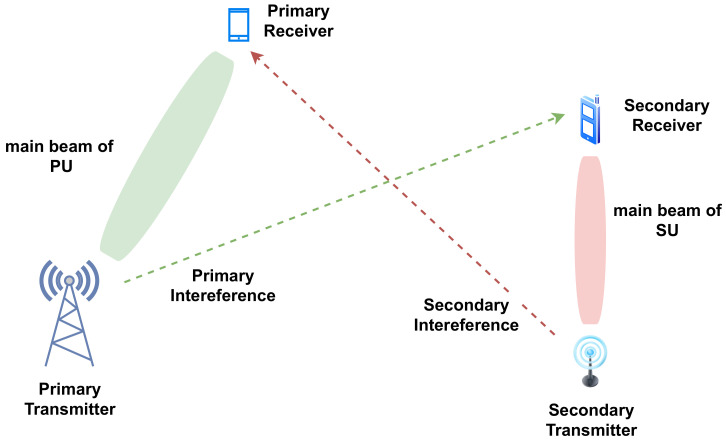
Spatial Dimension of CR application: SU is able to exploit the spatial dimension by transmitting in a non-overlapping direction with the PU transmission. Here, SU should be able to estimate/know the PU beam in order to avoid the interference.

Massive MIMO and Ultra-dense Network deployment are key enablers of 5G aiming at maximizing the spectrum reuse [196]. In this context, the base stations adopt tight beamforming techniques looking to maximize the transmit power in the direction of the receiver. Moreover. Cell-free networks have recently been proposed to serve a high number of users in a cellular network. A key enabler of this technology is the beamforming technique [197,198]. Here, CR may exploit a new dimension for its opportunistic transmission in addition to the time-frequency dimension: the spatial dimension. An example of new spatial dimension of CR is illustrated in Figure 5. SU becomes provided with the ability to transmit the data concurrently with PU but under the constraint that the SU transmits the data in a different beam than that of PU.

When the transmission beam of the PU is well identified, SU may exploit the remaining space in order to transmit the data on a non-overlapping beams base [125,199,200]. SUs, in this case, should be equipped with a multi-antenna system in order to adjust their beam far from the primary receiver to avoid interference. In this context, joint sensing and localization of PU becomes of high importance since the localization of PU transmitter may facilitate the mission of SUs in identifying the beam and thus to diagnose the spatio-temporal availability of the spectrum [200,201]. Recently, some work has been introduced to enable spatial SS by identifying the PU’s location and adjusting the SU’s beam using the received power at SUs surrounding the PU [200,202]. In this regard, estimation techniques have to be more developed in order to estimate the PU beam blindly or in the case where little information about PU transmission is available [203,204,205]. Nevertheless, identification of the PU transmission remains a complicated task for the SUs, especially where no cooperation is available between secondary and primary networks. In addition, interference caused by the secondary transmission should be carefully controlled since it is inevitable even though the transmission is beamforming-based. The interference level depends on the number of transmit antennas at the SUs, the distance between SU and PU and the angle of arrival of the secondary signal at the primary receiver. Accordingly, the transmit power and the beam of SU transmission should be adjusted.

## 7. Future Challenges

The main philosophy of CR is to dynamically share the spectrum among different wireless communication technologies. Accordingly, SS for CR is highly impacted by the advancement and growth of these technologies. Wireless communication is undoubted evolving in many ways including infrastructure deployment (e.g., massive deployment of small cells), system operation and management (e.g., self-organized networks), new concepts (e.g., cell-free systems), new technologies (e.g., intelligent reflecting surfaces) and many other techniques. Thus, SS should keep up with the evolution progress. In the following, we present several future challenges related to applying and developing SS for the new trends of CR.

1. Channel Coding for Interference Sensing:
  IS is mainly applied in the TR mode of the FDCR. Being able to use only one available channel to establish bidirectional communication between two SUs, TR becomes very attractive since it doubles the frequency efficiency compared to TS mode and HDCR [65,78]. TR uses signal decoding to reveal the PU status, which depends on the adopted channel coding technique. The weak technique may deteriorate the performance of the secondary network, while the strong technique may allow SU to decode the received signal even if PU is active. Here, the challenge becomes how to choose the optimal channel coding technique that matches the quality of service of SUs and, at the same time, does not prevent SU from detecting PU.
2. Switching protocols between CR functioning modes:
  Existing techniques for switching from a CR mode to another only take into account PU statistics [72,76,78]. However, other parameters may be taken into consideration such as the energy and frequency resources, since each mode has different requirements. Modes that are based on SIC, such as TS and TR, require more hardware and energy resources. This is not always available, especially for the battery-powered devices, which are planned to serve for several years such as the LPWAN IoT devices. The adoption of a mode depends on the available frequency resources: TR may be one of the good choices since it requires only one channel to establish bidirectional communication between two peer SUs, but it suffers poor sensing performance at low PU SNR. Thus, both frequency and energy efficiencies are important factors that should be taken into account to make a suitable choice of the mode to adopt. With this large number of parameters, learning techniques can be extremely useful to indicate the most suitable mode to adopt by the SUs.
3. Access Strategy for IoT/WSN networks:
  In IoT applications, contention between SUs is high due to the large number of sensors. Thus, the adopted spectrum sharing strategy in such application becomes of high importance to effectively manage the access of different types of sensors [206,207,208]. This strategy may be related to the data type to be sent by the sensor, the redundancy of the data (redundant data could be ignored or compressed) and the criticality. Sensors looking for transmitting critical data, especially those related to natural disasters and e-healthcare, may be prioritized over the other sensors. A strategy giving the sensors a weight is a common approach in WSN to alleviate interference [209]. Such a strategy may be useful in CR-IoT applications to manage the access of the nodes on the available frequency channels and maximize spectral efficiency.
4. Exchange Protocol of SS data for IoT/WSN
  Developing adequate protocols for CR-IoT systems is essential to manage the exchange between the central entity of the IoT network and the nodes [145,210,211]. This includes requests for nodes to sense a given channel and informing the concerned nodes with the channel availability updates. For sensing requests, the energy need of the IoT nodes should be highly considered especially when the nodes are battery-based. In this regard, selecting the sensors to sense the channel, the number of sensing processes per day and the maximal sensing observation time of the sensor should be determined by the central entity of the IoT/WSN network to ensure effective utilization of the resources. Moreover, the nodes that want to send data should be informed by the central entity about the available channels a priori. Thus, effective protocols should be designed to ensure the time and the frequency synchronization between the end-nodes and the central entity.
5. Use of Intelligent Reflecting Surfaces:
  SS may benefit from the emerging Intelligent Reflecting Surface (IRS), which is expected to play an essential role in 5G and B5G technologies [212,213]. IRS can passively reflect the signal towards a target receiver. IRS is a potential candidate to help to overcome the hidden PU problem by reflecting the PU signal towards the SUs, which suffer from low PU SNR. Several challenges are expected in using IRS to assist SS, since the optimal configuration of the IRS system depends on the channel between PU and IRS, IRS and SU, and PU and SU. In a context, where no cooperation is available between SU and PU, channel estimation becomes hard to apply. Blind channel estimation and cascaded-channel estimation could be a good candidate to help the IRS application for SS assistance [214].
6. Sensing the Spatial Dimension for CR
  Beam-based sensing of PUs becomes more and more important for SUs since it provides the SU with the spatial availability of the spectrum. However, the PU’s transmission beam estimation remains challenging for SU especially where no cooperation is available with PU [200,215]. Even when the PU beam is known, adjusting the SU beam is challenging too due to the inevitable interference caused by the SU transmitter to the SU receiver. Thus, the transmit power, the beam direction, and the number of transmit antennas should be carefully adjusted. However, due to the need for multiple antennas to adjust the SU beam, applying beam-based CR is challenging for low-cost IoT/WSN devices.
7. Towards Intelligent Spectrum Sensing:
  With the massive small cell deployment and Massive Machine-Type Communication in 5G and B5G, the binary decision of the SS may not be efficient. In such a deployment the SS output may be vulnerable to a high false alarm rate due to the inter-cell interference, i.e., a given channel is free in the cell where SU exists, but SU may falsely detect the presence of PU due to the inter-cell interference coming from another cell [216]. For this reason, a more intelligent and flexible SS technique should be adopted to overcome the homogeneity assumption of the PU coverage [129]. This means that the SU should be able to diagnose the channel as free even though PU is detected in some circumstances. Moreover, SS should be extended to deal with spectrum perception and environment dynamics learning. This is extremely important especially for battery-power devices to enable joint channel sensing and access.

## 8. Conclusions

In this survey, we presented the fundamental principles and motivations of applying spectrum sensing in cognitive radio networks. The concepts of half-duplex and full-duplex cognitive radio are presented. The main criteria exploited by SU to make the PU signal detection are presented and discussed. Different modes of operation for the case of full-duplex are described. Moreover, the use of learning techniques are discussed at both local and cooperative levels. Then, the potential of applying spectrum sensing in WSN/IoT network is investigated, in addition to the essential role of IoT/WSN in the Spectrum Sensing as a Service. We also discuss the use of cognitive radio in 5G and B5G from spectrum allocation and frequency efficiency perspectives. Based on exhaustive surveying of the state of the art, we present several challenges and staggering points that need to be further investigated.

## Figures and Tables

**Table 1:** List of abbreviations used in the survey.

Abbreviation	Definition
5G	Fifth Generation
ACD	Autocorrelation Detector
ADC	Analog to Digital Converter
B5G	Beyond 5G
CPSD	Cumulative Power Spectral Density
BS	Base Station
CR	Cognitive Radio
CS	Compressive Sensing
CSAT	Carrier Sensing Adaptive Transmission
CSD	Cyclo-Stationary Detector
DCS	Dynamic Channel Selection
DL	Deep Learning
DSA	Dynamic Spectrum Allocation
ED	Energy Detector
eMBB	enhanced Mobile Broad-Band
EVD	Eigen Value based Detector
FDCR	Full-Duplex Cognitive Radio
FC	Fusion Center
GoF	Goodness of Fit
HDCR	Half-Duplex Cognitive Radio
HSS	Hybrid Spectrum Sensing
IBFD	In-Band Full-Duplex
IS	Intereference Sensing
IoE	Internet of Everything
IoT	Internet of Things
IRS	Intelligent Reflecting Surface
LAT	Listen and Talk
LBT	Listen Before Talk
LPWAN	Low-Power Wide Area Network
LTE	Long Term Evolution
LTE-LAA	LTE-Licensed Assisted Access
LTE-U	LTE-Unlicensed
ML	Machine Learning
mMTC	Massive Machine-Type Communication
NU	Noise Uncertainty
OFDM	Orthogonal Frequency Multiple Access
PU	Primary User
RSI	Residual Self-Interference
SDR	Soft Defined Network
SI	Self-Interference
SIC	Self-Interference Cancellation
SS	Spectrum Sensing
SNIR	Signal to Noise and Interference Ratio
SNR	Signal to Noise Ratio
SSaas	Spectrum Sensing as a Service
SU	Secondary User
SVM	Support-Vector Machine
TR	Transmit-Receive
TS	Transmit-Sense
URLLC	Ultra Reliable and Low Latency Communication
WBS	Wide Band Sensing
WFD	Waveform Detector
WSN	Wireless Sensor Network

**Table 2:** Comparison among the widely used Spectrum Sensing detectors, with respect to: need for Secondary User (SU)–Primary User (PU) cooperation, the Noise Uncertainty (NU) effect, and the computational complexity, for a number of received samples *N*.

Detector	Requires PU-SU Cooperation?	Affected by NU?	Computational Complexity	Remarks
**ED**	No	Yes	2N−1	[30,46]
**Generalized ED**	No	Yes	Nf(p)	f(p) is related to the adopted power exponent. Please refer to [33]
**CSD**	Yes	No	$\begin{array}{c} (N_{s}-1)(N(L+1)+8N_{s}^{2}-\\ \;\;\;10N_{s}+4L^{2}+4) \end{array}$	*L* is an odd number and stands for the length of a unit window used in CSD [37,47,48,49]
**EVD**	No	No	$N_{s}^{2}KN$	*K* is the smoothing factor, $N_{s}$ is the oversampling factor [41,50]
**ACD**	No	No	$N_{s}\left(2N+1\right)$	$N_{s}$ is the oversampling factor [51,52]
**WFD**	Yes	No	M(2N−1)	*M* is the number of blocks used to evaluate the WFD [46]
**CPSD**	No	Yes	$N(3+\log_{2}\left(N\right))$	[29]
**Normalized CPSD**	No	No	$1+N(3+\log_{2}\left(N\right))$	[29]
**GoF**	No	No	2N	[42,43]

**Table 3:** Comparison among the CR operating modes in terms of reliability, collision time, need for SIC and the support of bidirectional communication.

Mode	Reliable SS at Low SNR	Collision Time	Needs SIC for Sensing	Bidirectional Communication	Notes
**HDCR**	Yes	Long	No	No	Classical HDCR does not have SIC circuit. Thus bidirectional communication and TS are not applicable [22,36,81].
**FDCR-TS**	Yes	Short	Yes	No	SIC is used to apply simultaneous Transmit-Sense strategy. No simultaneous bidirectional communication is applied in this mode [66,69,82,83].
**FDCR-TR-IS**	No	Short	No	Yes	SIC is used in this mode to establish bidirectional communication. The PU sensing is done based on the IS [65,76,77,78].
**FDCR-TR-SS**	Yes	Short	No [75,79]/Yes [80]	Yes	Even though SIC is used in this mode to apply simultaneous bidirectional communication, SS remains applicable with the help of ensuring a certain level of cooperation between the communicating SUs [75,79,80].

**Table 4:** Classification of recent papers that apply learning techniques in CR with respect to the covered topic. The mark **✓**(resp. **✗**) means that the subject is covered (resp. is not covered) by the mentioned papers.

Research Papers	Local SS	Cooperative SS	Spectrum Prediction	Resource Allocation
[95,97,98,99,100,119,120]	**✓**	**✗**	**✗**	**✗**
[14,20,90,91,93,121,122,123,124]	**✗**	**✓**	**✗**	**✗**
[85,89,103,104,106,125,126]	**✗**	**✗**	**✓**	**✗**
[102,108,110,111,127,128,129,130,131,132,133,134]	**✗**	**✗**	**✗**	**✓**
[105,107,135,136,137]	**✓**	**✗**	**✓**	**✗**
[101,138,139,140,141]	**✗**	**✓**	**✗**	**✓**
